# Evaluation of a combined strategy directed towards health-care professionals and patients with chronic obstructive pulmonary disease (COPD): Information and health education feedback for improving clinical monitoring and quality-of-life

**DOI:** 10.1186/1471-2458-9-442

**Published:** 2009-12-01

**Authors:** Carles Valero, Mònica Monteagudo, Maria Llagostera, Xavier Bayona, Sílvia Granollers, Mateo Acedo, Juan J Ferro, Lluïsa Rodríguez-Latre, Jesús Almeda, Laura Muñoz, The COPD Group of SAP Baix LLobregat Centre

**Affiliations:** 1 Unit Methodology, Assessment and Quality, SAP Dreta, Catalan Health Institute, 08025 Barcelona, Spain; 2 IDIAP Jordi Gol i Gurina, 08007 Barcelona, Spain; 3 Unit Assessment and Quality, SAP Baix Llobregat Centre, Catalan Health Institute, 08940 Cornellà de LLobregat, Spain; 4 SAP L'Hospitalet, Catalan Health Institute, 08901 L'Hospitalet de Llobregat, Spain; 5 ABS Sant Just Desvern, SAP Baix Llobregat Centre, Catalan Health Institute, 08960 Sant Just Desvern, Spain; 6 SAP Baix Llobregat Centre, Catalan Health Institute, 08940 Cornellà de LLobregat, Spain; 7 Unit Pharmacy, SAP Baix Llobregat Centre, Catalan Health Institute, 08940 Cornellà de LLobregat, Spain; 8 Unit Methodology, Assessment and Quality, SAP Baix Llobregat Centre, Catalan Health Institute, 08940 Cornellà de LLobregat, Spain; 9 Research Support Unit, DAP Costa de Ponent, Catalan Health Institute, IDIAP Jordi Gol, CIBER Epidemiology and Public Health (CIBERESP), 08907 L'Hospitalet de LLobregat, Spain; 10 Research Support Unit, DAP Metropolitana Nord, Catalan Health Institute, IDIAP Jordi Gol, 08303 Mataró, Spain

## Abstract

**Background:**

Chronic obstructive pulmonary disease (COPD) is a health problem that is becoming increasingly attended-to in Primary Care (PC). However, there is a scarcity of health-care programs and studies exploring the implementation of Clinical Practice Guidelines (CPG). The principal objective of the present study is to evaluate the effectiveness of a combined strategy directed towards health-care professionals and patients to improve the grade of clinical control and the quality-of-life (QoL) of the patients via a feedback on their state-of-health. A training plan for the health-care professionals is based on CPG and health education.

**Method/Design:**

Multi-centred, before-after, quasi experimental, prospective study involving an intervention group and a control group of individuals followed-up for 12 months. The patients receive attention from urban and semi-urban Primary Care Centres (PCC) within the administrative area of the Costa de Ponent (near Barcelona). All the pacients corresponding to the PCC of one sub-area were assigned to the intervention group and patients from the rest of sub-areas to the group control. The intervention includes providing data to the health-care professionals (clinician/nurse) derived from a clinical history and an interview. A course of training focused on aspects of CPG, motivational interview and health education (tobacco, inhalers, diet, physical exercise, physiotherapy). The sample random includes a total of 801 patients (≥ 40 years of age), recorded as having COPD, receiving attention in the PCC or at home, who have had at least one clinical visit, and who provided written informed consent to participation in the study. Data collected include socio-demographic characteristics, drug treatment, exacerbations and hospital admissions, evaluation of inhaler use, tobacco consumption and life-style and health-care resources consumed. The main endpoints are dyspnoea, according to the modified scale of the Medical Research Council (MRC) and the QoL, evaluated with the St George's Respiratory Questionnaire (SGRQ). The variables are obtained at the start and the end of the intervention. Information from follow-up visits focuses on the changes in life-style activities of the patient.

**Discussion:**

This study is conducted with the objective of generating evidence that shows that implementation of awareness programs directed towards health-care professionals as well as patients in the context of PC can produce an increase in the QoL and a decrease in the disease exacerbation, compared to standard clinical practice.

**Trial Registration:**

Clinical Trials.gov Identifier: NCT00922545;

## Background

Chronic obstructive pulmonary disease (COPD) is described as a disease of restricted air flow, not totally reversible and, in general, progressive. It is associated with an abnormal inflammatory response of the lung to toxic particles and gases, especially tobacco, with important systemic repercussions [1]

It is a very prevalent health problem worldwide and is a frequent cause of morbidity and mortality in developed countries. Studies conducted in Spain show a prevalence of between 8 and 12% in the population ≥ 40 years of age and 20% in those ≥ 65 years of age [2-5]. In our environment it represents a quarter of all-cause death, with an overall rate of 33 cases/100,000 individuals/year [6]. Based on several studies, the calculated worldwide level in the year 2020 will be the 5^th ^highest cause of invalidity and the 3^rd ^highest cause of death [7].

COPD is responsible for between 10-12% of the consultations in Primary Care (PC) and 35-40% of the consultations in specialist care. It is responsible for 7% of hospital admissions and 35% of chronic incapacity with respect to productive labour [6] and, as such, the social, health-care and economic impact is high. The cost of this disease accounts for 2% of the annual budget of the Ministry of Health and Consumer Affairs [6]. It is calculated that the patient with COPD generates a mean direct cost of 1,876 €/year; the greater part of the cost corresponding to the hospitalisation (43.8%) followed by drug costs (40.8%) for the control of the disease [8].

The approach to COPD, according to the different clinical guidelines [6,9] includes prevention, diagnosis, follow-up with pharmaceutical as well as non-pharmaceutical treatment, and the coordination between the PC and the health-care provision facilities (specialist care and hospitalisation).

The tobacco habit is the best known causal factor of the disease [9]. Smoking has a high prevalence of 29.5% in the general population [10] and of 80% in patients with COPD [11,12]. Cessation of smoking is the only measure that can prevent the disease and modify its clinical course [9,13]. Several studies indicate that, at the level of PC, there is low implementation of structured programs for the cessation of smoking [14,15]. Several questionnaires have shown that an important proportion of the patients with COPD do not identify smoking as the principal cause of their disease, and a high proportion had never attempted to quit smoking [16,17].

Several international studies [9,18-21] have shown that a high proportion of patients with COPD are non-diagnosed, including those in the advanced stages of the disease. In our environment, only 22% of the patients had been diagnosed previously [22,23]. The reasons for delay and of under-diagnosis are due, in part, to the lack of awareness by the patients and, as well, by their own family doctor. The term COPD appears not to be recognised nor understood by the general population. According to a study conducted in Spain, only 9% of the general population have some knowledge of COPD and, among those individuals with chronic symptoms and high risk of COPD, 33.2% had never consulted their general practitioner [24]. With respect to clinicians, there needs to be an increase in the awareness of COPD, especially at the PC level where, for example, there is a low level of spirometric testing [24]; a technique that is vital for the diagnosis of the disease. Recent studies indicate that only between 38 and 45% of the patients at high risk of COPD have had a spirometric test performed by the family doctor in the PCC [20,24-26].

In patient follow-up, it is important to highlight that clinical practice impacts on symptom control, essentially the dyspnoea, and this has a clear effect on the QoL of the patient [27] whereas other more objective parameters (such as spirometry data) are not always related so directly with this aspect. An objective measure of dyspnoea can be obtained with the modified scale of the Medical Research Council [28,29]. Questionnaires specifically related to COPD, and adapted for our environment, are the St George's Respiratory Questionnaire (SGRQ) [30,31] and the Chronic Respiratory Disease Questionnaire (CRQ) [32,33], and these are useful tools in evaluating the effectiveness of the interventions in these patients.

Clinical Practice Guidelines (CPG) advocate implementing health education programs during the follow-up of the disease. Educational interventions need to include aspects such as positive motivational reinforcement, anti-smoking advice, information on diet and physical exercise, compliance with the therapeutic regimen, and verification of the correct inhaler technique [6,7,9,34]. One of the most useful strategies in achieving a change in life-style habits is the motivational interview. However, this is conducted irregularly, and with little structure. In exploring the attitude of the patients in relation to their health-related life-style activities, the General Health Survey of the Cancer Prevention Research Center [35] is useful since it responds to the patient's intention towards the habit (to continue or to discontinue). This exploration enables an assessment to be made regarding the stage prior to the evolution of change which, according to Prochasha and Diclemente [36,37] can help establish a therapeutic plan. In our Spanish environment there is little tradition of health education programs for patients with COPD, while those that do exist focus on aspects of training in the use of inhalers and, in individual consultation, on the management of the smoking habit. It advised that respiratory rehabilitation be performed within the treatment plan for the patient with COPD [6] but this is precluded by the lack of sufficient numbers of health-care professionals, the time or adequate space in which to implement an appropriate level of service at the PC level and a low implementation of specific programs for this pathology, including a multi-disciplinary approach to the disease.

In relation to drug-based treatment, studies performed in Spain show that outpatient treatment of the disease in PC is far from the evidence-based recommendations for daily clinical practice [28].

In the real world of PC, the health-care professionals have a multitude of guidelines to follow and decisions are often taken that are highly variable. As such, a standardisation of CPG would be useful if disseminated and implemented correctly [38] using a well-regulated training plan. There are few studies that have explored the implementation of CPG in patients with COPD, not only nationally but internationally as well and, of these, the majority have evaluated only the pharmaceutical viewpoint [32,39].

Also needing highlighting is the importance of health-care professionals making clinical information available to their patients with COPD. Currently, there are only a few of these aspects that the teams of health-care professionals in the PCC make available to the patients. These include spirometry and vaccinations and, for the individual, the profiles of prescription drugs. There are not many pamphlets and leaflets available, nor are patients provided with individualised information and outcomes by their health-care professionals (control of symptoms, spirometry results, QoL) which can improve standard clinical practice. But there has been a change in the information provided on prescription medications and the use of diagnostic tests [40].

Hence, we developed the current project which focuses on evaluating the effectiveness of a combined strategy directed towards health-care professionals as well as the patients in order to improve the grade of clinical control/monitoring and the QoL of patients with COPD. The program involves individual patient health-status information feedback to their health-care professionals, a plan of training of health-care professionals based on a CPG [7] involving aspects of health education, and the implementation of the program when the patient visits the PCC.

The specific objectives are: a) to describe the current status of patients with COPD attending PCC. The evaluations include the principal socio-demographic variables, risk factors, prevalence, clinical monitoring and follow-up, the consumption of health-care resources, drugs, health advice and QoL; b) to evaluate the grade of compliance with the recommendations of the CPG [7] in monitoring COPD; and c) to evaluate the possible life-style changes (tobacco habit, diet and physical exercise) that may be spontaneous and/or taken-up following the implementation of the health-care intervention.

## Methods/Design

**Figure 1:**
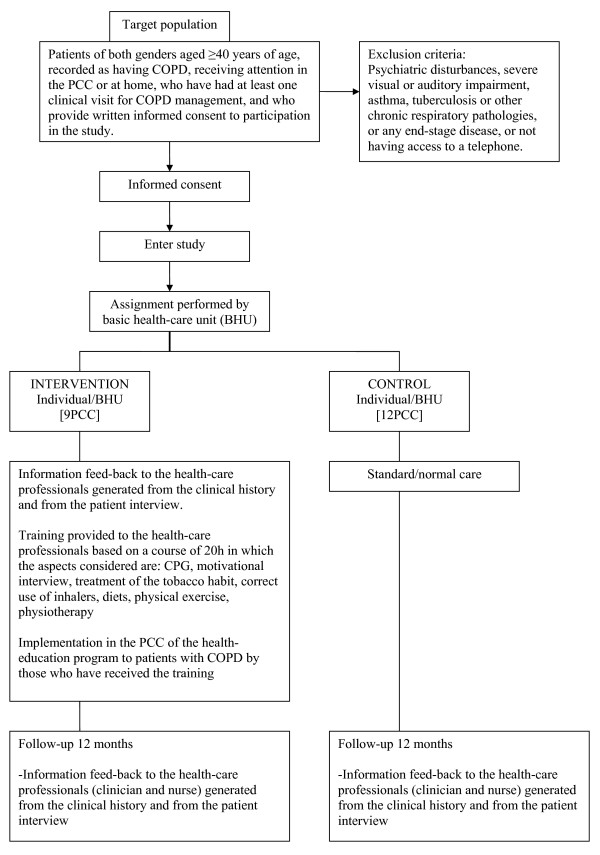
**Algorithm of the study**.

This is a multi-centred, before-after, quasi experimental, prospective study involving an intervention and a control group of individuals followed-up for 12 months (Figure 1).

Participation in the study was offered to all the urban and semi-urban PCC within our health-area administrative remit (52 PCC of which 21 took-up the invitation to participate). All the patients corresponding to the PCC of one sub-area (n = 9) (SAP Baix LLobregat Centre) were assigned to the intervention group and the patients from the rest of the PCCs (n = 21) in the administrative area (L'Hospitalet, Baix Llobregat Nord, Litoral i Alt Penédes-Garraf) were assigned to the control group

Given that this project is based on an overall strategy for the management of COPD involving a training program for health-care professionals of the PCC and, subsequently, the implementation of the program by the health-care professionals with their patients, there was no randomisation performed. This assignment was performed for logistic reasons and to maximise the benefit while minimising the contamination effect between PCCs. Since the PCCs are from the same metropolitan area of Barcelona, there are not expected to be any significant differences in terms of socio-economic and education levels that could affect outcomes.

### Study subjects

The subjects include all patients diagnosed as having COPD attending the PCC for the management of their pathology over the previous year. Inclusion criteria are: patients of both genders aged ≥ 40 years of age, recorded as having COPD, receiving attention in the PCC or at home, who have had at least one clinical visit for COPD management, and who provide written informed consent to participation in the study.

Exclusion criteria are: patients who have any psychiatric disturbances, severe visual or auditory impairment that would impede compliance with the study protocol, patients with asthma, tuberculosis or other chronic respiratory pathologies, or any end-stage disease, or not having access to a telephone.

### Sample selection process

The proposal to participate in the project was put to all the health-care professionals (clinicians and nurses) organised as basic health-care units (BHU); 87 BHUs corresponding to 21 PCCs participated in the study.

### Recruitment of patients

Since 2003, some centres had computerised their records while others have been in the process of computerisation but, currently, retain hardcopy (paper) clinical histories. Hence the recruitment is performed in different ways depending on the type of records being kept. In 8 centres that are not computerised, a list of patients ≥ 40 years of age was solicited from each participating BHU and, from which, the team investigator identifies the COPD patients from the clinical history. In 13 computerise centres, the team investigator solicits a list of the patients ≥ 40 years of age with the COPD diagnosis (Codes ICD-10: J43 or J44 or Codes ICPC-2: R79 or R95) in the database of the computerised clinical history (e-CAP or OMI-AP) from each participating BHU.

The BHU health-care professionals contact consecutive COPD patients via the telephone and, following the sequence on the list, solicit verbal consent to participation. Following the agreement to participation, an appointment is made for a clinical consultation at the PCC, or at the patient's home.

### Sample size calculations

The variable used for the calculation of sample size is the improvement in QoL according to the St. George's Respiratory Questionnaire (SGRO) with a score of 0 to 100. To detect a difference of ≥ 4.3 units between QoL scores of the two groups, the minimum number required would be 393 subjects in the control group and 393 patients in the intervention group. Assumptions are: a standard deviation value in the control group of 20.4 and the estimated rate of loss to follow-up of 10% [41,42]. The calculation has an α risk set at 0.05 and a β risk set at 0.20 in a two-tailed test.

### Intervention

The intervention consists of:

- Information feed-back to the health-care professionals (clinician and nurse) generated from the clinical history and from the patient interview

- Training provided to the health-care professionals based on a course of 20 h in which the aspects considered are: CPG, motivational interview, treatment of the tobacco habit, correct use of inhalers, diets, physical exercise, physiotherapy

- Implementation in the PCC of the health-education program to patients with COPD by those who have received the training

To be able to evaluate the efficacy of this intervention, the comparison is between the intervention group and the control group (formed of patients attended-to by the health-care professionals who did not attend the training course). The pre-intervention data collection is from the year 2004 and, after 1 year, the same information is collected post-intervention.

### Variables recorded

• QoL: variable collected using the SGRQ. This is a questionnaire designed to be used in diseases of obstructive airways, COPD and asthma [30], translated into Spanish [31]. It is self-administered and is composed of 76 items grouped on 3 scales or dimensions: symptoms, activity (activity that cause or are limited by dyspnoea) and impact (social functioning and psychological trauma caused by the respiratory disease). The scoring for each dimension and total score is from 0 to 100. High scores indicate a poor QoL and a change of 4.3 or more points is considered a significant change in the QoL of the patient.

• Dyspnoea: variable collected using the modified scale of the Medical Research Council (MRC) [28] with the following values:

0 = Absence of dyspnoea, except when performing intense exercise

1 = Dyspnoea on rapid walking or up a slight slope

2 = Inability to maintain, on a flat surface, the pace of other persons of the same age due to difficulty in breathing and requiring a stop to recover and to proceed at one's own pace

3 = The need to stop to rest when walking about 100 meters, or for a few minutes while walking at one's own pace

4 = Dyspnoea that impedes the patient going out of the home or in activities such as dressing and undressing

• Number of exacerbations and hospitalizations in the previous year

• Evaluation of the inhaler technique used by the patient: This is performed using a practical test involving evaluating the step-by-step inhalation technique according to the guidelines of the Spanish Society of Pneumology and Thoracic Surgery *[Sociedad Española de Neumología y Cirugía Torácica; SEPAR] *and the Spanish Society of Family and Community Medicine *[Sociedad Española de Medicina Familiar y Comunitaria; SEMFYC] *[43,44]. The technique is broken-down into several manoeuvres specific for the type of inhaler being used. One point is assigned for each manoeuvre correctly performed. A technique is considered to have been correctly performed if the score achieved is 9 or more [45].

• Stage assessed by spirometry [46]. The Forced Respiratory Volume in the first second (FEV1) is the spirometric parameter that is used to define the severity and to establish the functional classification [46] i.e.

Stage I slight: FEV1 ≥ 80%

Stage II moderate: 50% ≤ FEV1<80%

Stage III severe: 30% ≤ FEV1<50%

Stage IV very severe: FEV1 <30%

• Tobacco consumption:

- Never smoked

- Ex-smoker (one or more years since quitting smoking)

- Current smoker

### Other variables

• Socio-demographic: date of birth, gender, education level (no formal education, primary, secondary, university), country of origin, occupation over the last 10 years of employment according to the National Classification of Occupations [47].

• Risk factors: nicotine dependency according to the classification of Fagerström (high, medium, low), exposure (years) in the workplace (mines, textile, glass or plastic) and the environment (passive smoker, living close to a site of environmental contamination), and diseases associated with COPD (bronchial hypertrophy and atopia, respiratory infections in infancy and alpha-1 antitrypsin deficiency).

• Co-morbidity: arterial hypertension, diabetes mellitus II, dyslipidaemia, active cardiovascular disease, level of cardiovascular disease risk

• Life-style: regular exercise (a minimum of 23-30 minutes/day and 2-3 days/week), balanced diet (fruit and vegetables twice a day; bread, pasta, rice, other cereals and potatoes: 4-6 portions/day; cheese, yoghurt and milk: 2-4 potions/day; fish, chicken or eggs: 2 times/day; red meat, pork, sweet foods: 1 portion/week or less).

• Recorded at the time of the diagnosis: date (month and year) of the diagnosis of COPD, criteria of chronic bronchitis and emphysema.

• Tests solicited at the time of diagnosis: spirometry (FEV1 value and spirometry stage, chest X-ray, gasometry (PO_2_, Sat0_2_, CO_2_), electrocardiogram, blood analyses, weight and height and body mass index (BMI).

• Chronic symptomatology: presence of cough and sputum/expectoration over the past year

• Complications: presence of chronic *cor pulmonale*

• Characteristics of the exacerbations during the previous year: date, symptoms (dyspnoea, increase and purulence of sputum, fever); site of care received (PCC, emergency in PC, emergency in hospital); hospital stay; reason for not accessing PC attention (holiday or long waiting time at the PCC); treatment received (oxygen, oral corticotherapy, antibiotics, inhalers); need for secondary assistance; site of secondary assistance (PCC; PC emergency, hospital emergency); days admitted to hospital via own initiative or referral from PC.

• Care resources used in the previous year: PC consultations (outpatient and/or home based); specialist attention (public/private); rehabilitation services and physiotherapy

• Number of visits recorded in the clinical history in the previous year performed by: the doctor, nurse, specialist pneumologist

• Clinical opinion (pneumologist): doubts in the diagnosis, poor response to treatment, COPD moderate or severe, indication for home-based continuous oxygenotherapy (HCO), indication for volume reduction surgery or transplant, diagnosis of emphysema in patients >45 years of age, suspicion of obstructive apnoea syndrome (OAS) during sleep.

• Complementary tests solicited in the previous 2 years and in the follow-up of the patients with COPD: spirometry (FEV1 value in the clinical history or spirometry) gasometry (PO_2_, SatO_2_, CO_2_), haematocrit, electrocardiogram and body mass index calculation (BMI, weight and height); date of the measurements conducted.

• Preventive measures performed in the previous year. Advice on health education: anti-smoking advice, treatment of the tobacco habit (substitutes for nicotine), diet, physical exercise, complementary treatment, workplace and environmental exposure; immunisation (influenza and pneumococcus over the previous 5 years).

• Pharmacological treatment: Active prescriptions indicated for all the pathologies (listed by commercial name and number). Active prescription with indication for COPD:

➢ Bronchodilators:

- β2 short acting adrenergic inhalators: Salbutamol, Terbutaline, Fenoterol, Procaterol

- β2 long acting adrenergic inhalators: Salmeterol, Formoterol

-Anticolinergic inhalators: Ipratropium, Tiotropium

- Methylxantins: Theophylline, Etamiphylline

➢ Glucocorticoids (corticoids inhalators: Budesonide, Beclometasone, Fluticasone)

➢ Oral corticoid: Prednisone

➢ Other treatments for COPD:

- Antileukotrienes (Montelukast, Zafirlukast)

- Anti-inflammatories (Cromoglicate, Nedocromil, Ketotifen),

- Oral β2 adrenergics (Bambuterol, Clembuterol, Fenoterol, Salbutamol, Terbutaline)

- Mucolytics as standard treatment (Acetilcisteine, Ambroxol, Bromhexine, Carbocisteine)

- Antibiotics as COPD prophylaxis

- Home-based oxygenotherapy

• Complimentary therapy (difficulty in taking the medication and causes):

▪ I forgot to take the medication, I thought it was better not to take it

▪ I am very tired and do take the medication

▪ Because I did not pick-up the medication from the pharmacy

▪ I quit taking the medication because of side-effects

▪ I quit taking the medication because I felt better

▪ Because I am afraid of taking medications

▪ Because it is very expensive

▪ I just didn't take the medication

• Other questions

▪ Did you sometimes forget the inhaler or medications indicated for the treatment of your COPD?

▪ Do you take them at the times indicated?

▪ When you felt better did you quit the medication?

▪ Did you feel poorly when taking the medication and you decided to stop taking it?

• Inhalers: Number of inhalers and type of system used (MDI; MDI+ chamber; Autohaler, Aerolizer, Handyhaler, Turbohaler, Accuhaler)

• Attendance by the patient at the health-education classes. Follow-up variables: date, type of visit (programmed for the individual, programmed for the group, non-programmed)

• Discharge or withdrawn from the study (reasons): death, change of address, lost to follow-up, others

### Variables collected in follow-up (structured in 5 sections)

• Tobacco (diagnosis and intervention):

- Diagnosis (not evaluated, non-smoker, ex-smoker, smoker in the phase of pre-contemplation/contemplation/preparation, action/maintenance/relapse)

- Intervention (treatment in PC and follow-up, no intervention needed and the patient is congratulated, advice or information, motivational interview, minimal help, intensive help visits 7 days pre-intervention, visits 2-3 days post-intervention, visit 10-15 days post-intervention, visit in follow-up at 2-3 months).

• Inhaler use (diagnosis and intervention):

- Diagnosis (not evaluated, no prescription, prescription and not used, prescription used correctly, prescription not used correctly)

- Intervention (no intervention needed and the patient is congratulated, advice or information, motivational interview).

• Exercise (diagnosis and intervention):

- Diagnosis (not evaluated, not able to perform exercise, not performing exercise in the phase: pre-contemplation/preparation/action/relapse/performing exercise 20-30 minutes/day 2-3 days/week, performing exercise in the maintenance phase)

- Intervention (no intervention needed and the patient is congratulated, advice or information, motivational interview, personalised exercise plan).

• Diet (diagnostics and intervention):

- Diagnosis (not evaluated, healthy dieting, healthy diet in maintenance stage, non-dieting in phase of: pre-contemplation/contemplation/preparation/active/relapse)

- Intervention (no intervention needed and the patient is congratulated, advice or information, motivational interview, individualised diet)

• Respiratory physiotherapy: stretching exercise, seated exercise, bronchial hygiene, climbing stairs - (diagnosis and intervention evaluated in all the above exercises):

- Diagnosis (not evaluated, not having been shown, cannot perform exercise, exercise correctly performed, exercise incorrectly performed)

- Intervention (no intervention needed and the patient is congratulated, advice or information, motivational interview).

### Observations of the health-care professional

#### Collection of data and follow-up

In the patients from whom verbal consent to participation in the study has been obtained, the data are collected in a dual manner: clinical history (recorded, computerized or on paper) followed by an interview with the patient. This is performed in the PCC or at the patient's home. Written informed consent to participation in the study is then obtained. To minimise bias, the recording of the information (clinical history/interview) is conducted by the health-care professionals in the research team that has previously-received instructions in standardised data collection. The variables recorded in the clinical history and in the interview are recorded again at the end of the intervention period, one year later (Table 1)

**Table 1:** Distribution of variables according to the source and time of data collection.

	Variables source		Time of data collection	
	**Clinical** **history**	**Interview**	**Pre-intervention**	**Post-intervention**

Principal variables				

Quality of life; SGRQ		x	x	x
Evaluation of dyspnoea; MRC		x	x	x
Number of exacerbations	x	x	x	x
Number of hospital stays	x	x	x	x
Correct inhalation technique		x	x	x
Spirometry staging	x		x	x
Tobacco consumption	x	x	x	x

Other variables				

Date of birth	x		x	
Gender	x		x	
Education level		x	x	
Country of birth		x	x	
Occupation during the previous 10 years		x	x	x
Nicotine dependence		x	x	x
Work-place exposure		x	x	x
Environmental exposure		x	x	x
Disease related to COPD	x		x	
Co-morbidity	x		x	x
Life-style		x	x	x
Date of COPD diagnosis	x		x	
Chronic bronchitis and emphysema	x		x	
Co-morbidity at time of diagnosis	x		x	
Chronic symptomatology		x	x	x
Cor pulmonale	x		x	x
Characteristics of the exacerbations	x	x	x	x
Health-care resources used		x	x	x
Number of visits to the clinician/nurse/specialist, and reasons	x		x	x
Tests solicited over the previous 2 years	x		x	x
Preventative activities in the previous year	x		x	x
Drug treatment	x		x	x
Compliance with treatment (difficulties and causes)	x	x	x	x
Inhalers		x	x	x

The information is transferred to a specifically designed data-collection form and subsequently introduced into a database (Access version XP 2003) by a member of the administrative staff for subsequent validation and analyses.

The reference health-care professional (clinician or nurse) of the intervention group, following the feedback from their COPD patients and having attended the training program, interactively provide the health-education information to the patients. These consultations are monitored via a specific data-recording form (follow-up sheet) that is completed each time the health-education program information is provided by the clinician or the nurse to the patient, whether in a scheduled or a spontaneous visit to the PCC. Changes in stage (pre-contemplative/contemplative/preparative/action/relapse) of the life-style habits (tobacco, diet, exercise, respiratory physiotherapy, inhalator use) observed in the patient are recorded according to theoretical model of Prochaska and Diclemente (no intervention needed and the patient is congratulated/advice or information/motivational interview/individualised plan).

These data sheets are submitted to the project investigators on a monthly basis.

The numbers of visits that each patient needs for health education are determined depending on the level of change observed in the patients (taking into account the initial status of the individual, the motivation for change in conduct and the capacity to learn).

### Analysis strategy

Initially, the descriptive statistics are calculated for all the variables considered. These are the mean (standard deviation) for the continuous variables with normal distribution, the median (interquartile ranges) in case of non-normal distribution and frequency (percentage) for the categorical variables. The differences between groups (control vs. intervention) are evaluated using the χ^2 ^test or Fisher's exact test for the categorical variables and the Student *t*-test for the continuous variables with normal distribution, or the corresponding non-parametric Mann-Whitney U test.

The differences between means, medians or differences in proportions, at baseline and at the year-end (pre- and post-intervention) are compared using the appropriate McNemar test, the Student *t*-test for paired observations, or the Wilcoxon signed rank test. An intent-to-treat analysis is performed, followed by a second analysis with only those patients who had completed the study i.e. 1 year of follow-up. Results on prevalence are derived from the intent-to-treat analysis.

To evaluate the degree of change pre- and post-intervention, the differences in means between continuous variable values at baseline and post-intervention are adjusted for the size effect. The size effect is calculated as the difference between the mean at baseline and the measure at the end of follow-up divided by the baseline standard deviation value. This approximation is considered the size effect and is a standard measure of the change in a "before-after" study [48]. As defined by Cohen, a size effect of 0.20 is considered small, 0.50 as moderate, and 0.80 as large [49]. Analysis of covariance adjusted for the baseline values are performed as well.

The percentage change relative to baseline is used as a measure of change pre- and post-intervention in the categorical variables and is presented as the difference between the final percentage minus the initial percentage × 100.

Multivariate regression analyses are performed using the intervention/control group as dependent variable with the possible independent predictive variables and those that are considered clinically relevant adjusted for the baseline values. The level of significance is set at p < 0.05. The SPSS package, version 16.0 is used throughout

### Ethical aspects of the study

The protocol has been studied and approved by the CEIC -*Comité Ético de Investigación Clínica *(Clinical Investigation Ethical Committee) of the *Institut d'Investigació Primària Jordi Gol *[IDIAP-Jordi Gol]. Consent is obtained from the health-care professionals and from the patients for the collection of clinical history data. Data confidentiality of the patients and of the participating health-care professionals are maintained at all times. The data are used only for the scientific purposes of the study and anonymity is guaranteed in the presentation of the results.

## Discussion

### Limitations of the study

A major bias of the study is that there is no randomisation of the health-care professionals and of the participating centres. This is a quasi-experimental study in which the investigators control the exposure variable, but not the randomisation variable, and this can affect the external validity. Another possible bias is the restrictive criteria of patient selection. Included in the study are patients who can cope with an interview, even at home. Conversely, not included are those patients who, at the time of the interview, are unable to respond to the questions. This can affect the generalisability of the results.

One aspect needing to be taken into account is that the study does not compare whether the diagnosis of COPD has been done correctly. In recent years in PC, the diagnosis and management of COPD has been considered deficient. Recent studies indicate that only between 38 and 45% of patients at high risk have had a diagnostic spirometric analysis performed by the clinician in the PCC [20,24-26]. The most frequent explanations for this low percentage are: the lack of awareness of the impact of the test, unfamiliarity, lack of training, shortage of personnel and time, and the difficulty in interpreting the test [26,50,51]. European studies such as that by Rutschmann et al have shown that 82% of the clinicians in PC consider spirometry as the most appropriate method for the diagnosis of COPD while only 55% of them explicitly use the test and only 33% know the correct GOLD diagnostic criteria [14].

Of note is the low availability of the spirometric equipment in the PCC. Until quite recently the equipment was available in only 50% of PCCs [50,51]. Also, there is the lack of training of the technician in charge of performing the test and of maintaining regular quality control checks on the equipment. Even when these checks have been done, early studies showed a low level of quality control [52].

Hence, we decided to include patients whose clinical history notes included the term COPD in the diagnosis section without confirming whether or not spirometric testing had been performed, or that the result of the test corresponded to the obstructive pattern. This is acceptable within the framework of the present study because the objective of the study is not to validate the diagnosis but to evaluate the characteristics of the standard care provided in the PCC by the health-care professionals, and the response to an intervention program.

Another aspect warranting comment is that in this study only a single spirometric value was derived, the FEV1. This is because this is the most-frequently registered value in the clinical history of these patients and indicates the severity stage of the disease. As has been described in other studies which refer to the interpretation of spirometry results, apart from FEV1 all the remaining parameters show wide variation with a high percentage of values lying beyond the intervals considered plausible. Hence, the FEV1 is considered more reliable than the other parameters [53,54].

The present study provides us with a global as well as a detailed view of the treatment and follow-up of the patients with COPD receiving attention in PC. However, the results will need to be compared with other comparable studies.

### Applicability in the clinic

The study provides insight into standard clinical practice of the health-care professionals in relation to patients with COPD receiving attention in PC. At the same time it enables us to evaluate the possible changes that can appear naturally and, following an intervention, in the management of these patients and, consequently, to evaluate the appropriateness of these recommendations in CPG. As such, the study can have a special relevance since the majority of the studies previously conducted have focused on treatment with pharmaceutical products. The current study, on the other hand, attempts to provide a more global view of the correct management of patients with COPD. The results of the study would highlight the need for the correct application of diagnostic criteria that are currently recommended by CPG.

The smoking habit is, currently, the best known causal factor and the cessation of the habit is the only method that can prevent the disease and to modify its clinical evolution. Intervention in the PCC with respect to this risk factor is fundamental given that the health-care professionals, especially nurses, are very accessible to the public not only in the PCC but also in the community. As such, they represent effective instruments to motivate and to follow-up more intensively those patients who smoke. However, according to our literature search, the percentage of success in anti-tobacco programs is low. We consider it vital that the management of the smoking habit is broadened by encouraging the health-care professionals towards intervention by improving their training and awareness of the disease. This is an aspect the current project seeks to address.

In PC outpatient clinics, especially those managed by nursing staff, much effort is dedicated towards a few chronic pathologies such as diabetes, hypertension and obesity, while lacking a focus on other groups of patients with other chronic pathologies which could also benefit from such efforts.

The literature shows that non-pharmacological treatment, such as exercise and rehabilitation, can be useful in improving the exercise tolerance of the patients with COPD, essentially with respect to dyspnoea which is the symptom that most affects the QoL of the patient.

This project will confirm the need to support the workers in PC in attending to their patients with COPD in a programmed manner, to conduct health-education programs with the objective of improving the symptomatology, to diminish exacerbations, and to diminish the consumption of prescription drugs and health-care resources.

This project provides an overview of interventions that can improve the overall quality-of-life of the patients with COPD. Conduct modification or resource increases are needed when motivating the health-care professionals who, perhaps, are not fully aware of the overall health-care status of their patients. As such, we consider it of considerable value to inform the health-care professionals of those aspects that are susceptible to modification based on the information received from their outpatient clinics.

## Abbreviations

BHU: Basic Health-Care Units; COPD: Chronic Obstructive Pulmonary Disease; CPG: Clinical Practice Guidelines; CRQ: Chronic Respiratory Disease Questionnaire; FEV_1_: Forced Expiratory Volume in one second; GOLD: Global Iniciative for Chronic Obstructive Lung Disease; ICD: International Classification of Diseases; ICPC: International Classification of Primary Care; MRC: Medical Research Council; PC: Primary Care; PCC: Primary Care Centre; QoL: Quality of Life; SGRQ: St George's Respiratory Questionnaire.

## Competing interests

The authors declare that they have no competing interests.

## Authors' contributions

ML is the principal investigator and coordinator of the project. Design of the study was by CV, MM, ML, XB and LR-L. These authors, with the help of SG and MA designed the intervention and health-care education program. ML and SG designed and coordinated the training course of the CPG that was offered to the health-care professionals in the intervention group. MM, MA, JA and LM performed the statistical analyses. ML, MM and CV coordinated and presented the results (feedback) to the participating investigators in the intervention group. All the authors have read and revised the various versions of the manuscript and have approved this final version.

## Pre-publication history

The pre-publication history for this paper can be accessed here:

http://www.biomedcentral.com/1471-2458/9/442/prepub
